# Sorbent Film-Coated Passive Samplers for Explosives Vapour Detection Part A: Materials Optimisation and Integration with Analytical Technologies

**DOI:** 10.1038/s41598-018-24244-y

**Published:** 2018-04-11

**Authors:** Gillian L. McEneff, Bronagh Murphy, Tony Webb, Dan Wood, Rachel Irlam, Jim Mills, David Green, Leon P. Barron

**Affiliations:** 1 King’s Forensics, School of Population Health & Environmental Sciences, Faculty of Life Sciences & Medicine, King’s College London, London, SE1 9NH United Kingdom; 2 Threat Mitigation Technologies, Metropolitan Police Service, 113 Grove Park, London, SE5 8LE United Kingdom; 3 Air Monitors Ltd., 2/3 Miller Court, Severn Drive, Tewkesbury, Gloucestershire GL20 8DN United Kingdom

## Abstract

A new thin-film passive sampler is presented as a low resource dependent and discrete continuous monitoring solution for explosives-related vapours. Using 15 mid-high vapour pressure explosives-related compounds as probes, combinations of four thermally stable substrates and six film-based sorbents were evaluated. Meta-aramid and phenylene oxide-based materials showed the best recoveries from small voids (~70%). Analysis was performed using liquid chromatography-high resolution accurate mass spectrometry which also enabled tentative identification of new targets from the acquired data. Preliminary uptake kinetics experiments revealed plateau concentrations on the device were reached between 3–5 days. Compounds used in improvised explosive devices, such as triacetone triperoxide, were detected within 1 hour and were stably retained by the sampler for up to 7 days. Sampler performance was consistent for 22 months after manufacture. Lastly, its direct integration with currently in-service explosives screening equipment including ion mobility spectrometry and thermal desorption mass spectrometry is presented. Following exposure to several open environments and targeted interferences, sampler performance was subsequently assessed and potential interferences identified. High-security building and area monitoring for concealed explosives using such cost-effective and discrete passive samplers can add extra assurance to search routines while minimising any additional burden on personnel or everyday site operation.

## Introduction

The range of existing and emergent explosive types used in terrorist attacks worldwide has made it necessary for law enforcement agencies to continuously develop and enhance defensive search techniques and detection methods. High vapour pressure explosives evaporate readily making particulate detection challenging, including some of those used recently in improvised explosive devices (IEDs), such as peroxide-based materials^1^. The probability of identifying such concealed devices via traditional swabbing methods is very low, as it requires both efficient trace recovery and persistence of volatile particulate residues on the swabbed surface until analysis can be performed. Detection of the vapours emitted from concealed devices is sometimes a more successful and less time-consuming approach, and for this reason, vapour sampling and analysis is often performed in parallel during regular defensive searches of high profile targets.

A large variation exists in reported explosives vapour pressure data in the literature. A recent review paper by Ewing *et al*. consolidated all available data on explosives, excluded any outlier results and presented averaged vapour pressures in atmospheres normalised to 25 °C^2^. Primary explosives such as triacetone triperoxide (TATP) has one of the highest vapour pressures (~10^−5^ atm at 25 °C) and traces readily evaporate from surfaces over relatively short periods of time, potentially making it easier to detect in the vapour phase. In contrast, components of plastic explosives such as cyclotrimethylene-trinitramine (or “Research Department Explosive” (RDX)) and pentaerythritol tetranitrate (PETN) have very low vapour pressures (≤10^−11^ atm at 25 °C), making them difficult to detect in the vapour phase. In order to aid the detection of plastic explosives in the vapour phase, the International Civil Aviation Organisation (ICAO) requires the inclusion of high vapour pressure marking agents such as ethylene glycol dinitrate (EGDN), 2,3-dimethyl-2,3-dinitrobutane (DMNB) or 4-nitrotoluene (4-NT)^3^.

Analytical techniques, such as gas chromatography (GC), liquid chromatography (LC), capillary electrophoresis (CE), mass spectrometry (MS) and ion mobility spectrometry (IMS) are commonly used individually or in combination for the detection of trace explosive components^4–8^. The application of some of these techniques for explosives detection will be discussed in more detail in Part B of this paper, but for this section, we will focus on the various sampling techniques utilised for explosives detection in air. Sufficient sensitivity for trace vapour detection requires efficient sampling techniques, and in some cases, the inclusion of a pre-concentration step. Both passive and active sampling techniques have been used extensively in environmental monitoring studies, particularly for volatile organic compounds (VOCs) in air. Unlike active sampling which requires a power source, moving mechanical parts and regular maintenance, passive sampling is cheaper, easier, and more discrete for collecting air samples over longer periods and may minimise the need for high-frequency retesting^9^. As such systems function either by chemical absorption or physical adsorption of the vapour of interest onto or into the sampling medium, key factors to be considered include sampler specificity, capacity and quantitative potential^10^. Solid-phase micro-extraction (SPME) is a common sampling technique for the extraction and concentration of volatile and semi-volatile compounds and has been previously utilised for the passive uptake of explosive vapours^5,11^. More recently, planar solid-phase micro-extraction (PSPME) has been developed to further increase sampler surface area, thus increasing capacity and method sensitivity, and has been applied to both static and dynamic sampling of explosives in air^12,13^. With direct IMS analysis, TATP was previously detected within 30 seconds under static and dynamic PSPME conditions^12^ and dynamic PSPME sampling of headspace exposed to Pentolite and smokeless powders resulted in the detection of trinitrotoluene (TNT) and 2,4-dinitrotoluene (2,4-DNT), respectively^14^. For these described PSPME studies, glass fibre filters were coated with sol-gel polydimethylsiloxane (PDMS) nanoparticles which acted as a hydrophobic membrane and were used to selectively collect volatile analytes of interest while also acting as a barrier against external interferences. Active samplers have often used Tenax TA (polydiphenylphenylene oxide, PPPO) and Tenax GR (polydiphenylphenylene oxide with graphite, PPPO-GR) as a vapour pre-concentrator for explosives such as TNT and DMNB in occupational health exposure studies^15–18^. Thin layers of PPPO have recently been investigated as planar-type pre-concentrators for a small number of marking agents used in plastic explosives such as 4-NT and DMNB (also via active sampling in combination with subsequent GC-MS analysis)^19^. More recently, PPPO has been coated on micro pre-concentrators as an adsorbent solution for the selective pre-concentration of 2,4-DNT with sensitivity measured at mid ppb level^20^. However, even though the adsorbent properties of PPPO are well known, more knowledge is still required regarding its specificity, capacity, efficiency and uptake kinetics as thin films when applied to a wider range of explosives, precursors and transformation products in the vapour phase and in the presence of realistic levels of environmental interferences. This is also the case for other potentially suitable sorbent chemistries for use in passive sampling formats as well as suitable substrates for direct integration with explosive detection instrumentation, ideally to reduce the level of sample pre-treatment involved.

The aim of this work was to develop a passive air sampler for the qualitative detection of a wider range of volatile explosives-related components using thin film sorbents on chemically and thermally stable substrates. As shown in Table 1, explosives range from mid to high vapour pressures (10^−4^–10^−9^ atm at 25 °C) and this study focussed on 15 nitroaromatics, nitroesters, peroxides and marking agents. The film chemistry, including a range of sorbent types and film compositions, as well as a selection of chemically and thermally stable substrates were investigated with respect to uptake kinetics, recovery and stability in laboratory based studies. The ability to directly integrate planar sampler formats (i.e. with little or no sample preparation) with currently in-service explosives detection equipment including IMS and thermal desorption-mass spectrometry (TDMS) was also investigated in simulated exposure studies. With less associated consumables and staffing costs in comparison to traditional vapour sampling methods, such a device could be deployed autonomously over long exposure periods making it suitable for regularly searched buildings and areas.

**Table 1:** Molecular formula and weight, chemical structure and vapour pressure of selected explosive components.

Compound	Molecular formula	Structure	Molecular weight (g.mol^−1^)	Vapour pressure (atm at 25 °C)	Ref.
DADP	C_6_H_12_O_4_		148.0736	2.4 × 10^−4^	^2^
2-NT	C_7_H_7_NO_2_		137.0477	1.89 × 10^−4^	^37^
3-NT	C_7_H_7_NO_2_		137.0477	1.32 × 10^−4a^	
EGDN	C_2_H_4_N_2_O_6_		152.0069	1.02 × 10^−4^	^2^
4-NT	C_7_H_7_NO_2_		137.0477	6.43 × 10^−5^	^37^
TATP	C_9_H_18_O_6_		222.1103	6.31 × 10^−5^	^2^
DMNB	C_6_H_12_N_2_O_4_		176.0797	1.39 × 10^−5^	^2^
2,6-DNT	C_7_H_6_N_2_O_4_		182.0328	8.93 × 10^−7^	^2^
NG	C_3_H_5_N_3_O_9_		227.0026	6.45 × 10^−7^	^2^
2,4-DNT	C_7_H_6_N_2_O_4_		182.0328	4.11 × 10^−7^	^2^
2,3-DNT	C_7_H_6_N_2_O_4_		182.0328	∼10^−7b^	
3,4-DNT	C_7_H_6_N_2_O_4_		182.0328	∼10^−7b^	
HMTD	C_6_H_12_N_2_O_6_		208.0695	Σ10^−6^–10^−7^	^33,38^
TNT	C_7_H_5_N_3_O_6_		227.0178	9.15 × 10^−9^	^2^
HMDD	C_6_H_12_N_2_O_4_		176.0797	Not found	

^a^Predicted by ACD/Labs Percepta software.

^b^Estimated value based on vapour pressure of isomers 2,4-DNT and 2,6-DNT.

## Results and Discussion

### Quantitative performance of LC-UV and LC-HRMS methods

Compatible detection techniques with high sensitivity and selectivity are necessary to achieve low limits of detection (LODs) when used with passive samplers. For qualitative screening of explosives for threat mitigation purposes, techniques such as IMS and TD-MS are suitable. However, quantitative analytical techniques such as LC-UV and liquid chromatography-high resolution mass spectrometry (LC-HRMS) were required here for the development, characterisation and performance assessment of this new passive sampler. LC-UV method performance data can be found in Table S1 of the supplementary information. A significant advantage of using LC-HRMS for the detection of explosives lies in the ability to perform post-acquisition data-mining for potentially new or emerging compounds not present in this target analyte set. The mobile phase buffer (ammonium acetate and ammonium chloride) and sample injection volume (5, 10, 15, 20 µL) were optimised to obtain the greatest overall response for analytes in both positive and negative modes. A 0.2 mM ammonium chloride solution in a mixture of methanol and water and a sample injection volume of 5 µL produced the greatest response for most analytes, particularly those detected in negative ionisation mode, as previously observed in the LC-HRMS method utilised by Rapp-Wright *et al*.^4^. Performance was assessed here for all 15 selected analytes and this underpinned measurement of kinetics, recovery and particularly its application in field trials. Fourteen analytes were detected using HRMS under direct infusion. No ions were detected for 3-NT at 5 µg mL^−1^ (optimised parameters listed in Table S2). LC-HRMS instrumental and method performance results for retention time, m/z accuracy, precision, reproducibility, linearity, LOD, limit of quantification (LOQ), and range are shown for the full list of analytes in Table 2. Here, method performance included passive uptake by the finalised sampler design (Nomex substrate coated in a PPPO film) in the testing void (0.135 dm^3^ Duran glass bottles) and subsequent analysis. Analyte retention times varied ≤1% for both instrumental and method validation. Signal reproducibility for both peak area and peak height was assessed and signal height was found to show lower variability for most analytes following injection of standards and extracted samplers pre-exposed to vapours (n = 6). Peak height was used to quantify analyte signals due to the presence of conformers (for TATP only) and several isobaric analytes, such as DNT and NT-based compounds, which were not completely resolved chromatographically. Instrumental signal precision varied <30% for n = 6 standard replicates and similar results were found for method validation between replicate samples (n = 6) except for nitroaromatic compounds, which varied up to 47%, and hexamethylene diperoxide diamine (HMDD) which varied up to 67% (this compound was discontinued by the supplier due to instability issues in solution). For linearity and range, coefficients of determination (*R*^2^) were assessed for all compounds at concentrations from 1 ng mL^−1^ –100 µg mL^−1^ for standards and from 50–10,000 ng for vapour-exposed samplers. *R*^2^ values for instrumental and method validation were determined at >0.99 and >0.93, respectively, showing that the finalised sampler could be used for quantitative measurements, assuming some form of calibration can be performed *in situ*. Signals were found to be linear up to 250 and 500 ng on column except for diacetone diperoxide (DADP) which was only found to be linear up to 50 ng on column. LOD and LOQ values were determined at low pg level (standards injected directly on column) for TNT and DNT compounds and mid-high pg level for all other analytes, except 3-NT. For method validation, sampler sensitivity (passive sampling with LC-HRMS analysis) was high for nitroaromatic compounds at ≤10 ppt detected on sampler following vapour exposure but nitrate esters, nitrotoluenes and hexamethylene triperoxide diamine (HMTD) measured at low ppb levels. The detection limits shown in Table 2 are in the ng-µg dm^−3^ range when using LC-HRMS analysis which represents part per trillion-billion levels. As different detectors vary in sensitivity, LC-HRMS analysis was found to be particularly sensitive for nitroaromatic compounds however TD-MS analysis was found to be more sensitive for the nitroester compounds when used on samplers exposed during the application trials^21^.

**Table 2:** Passive sampling and LC-HRMS method and instrumental performance for the 15 selected analyte vapours.

Compound	*t*_r_ in min (%RSD) n = 6^a^	Calculated ion (m/z)	*δ* (ppm)	Recovery ± SD (%) n = 6^b^	Precision (height %RSD) n ≥ 6^a^		Linearity (*R*) n ≥ 9^c^		LOD n ≥ 9^c^		Linear range: Passive Sampler + LC-HRMS (ng on sampler) n ≥ 9^d^
					LC-HRMS	Passive Sampling + LC-HRMS	LC-HRMS^c^	Passive Sampler + LC-HRMS^d^	LC-HRMS (pg on column)^c^	Passive Sampler + LC-HRMS (ng dm^−3^)^d^	
HMTD	2.4 (0.7)	207.0976	−0.97	2 ± 1	7	30.4	0.999	n.d.	135	37000	n.d.
EGDN	2.9 (0.5)	61.9884	+1.61	93 ± 7	20	27.8	0.998	0.997	520	2000	2.5–50
HMDD	3.7 (0.7)	177.0870	−0.56	61 ± 35	24	64.9	0.993	0.998	270	370	0.5–100
3,4-DNT	4.8 (0.3)	182.0333	−0.55	79 ± 11	22	36.4	0.999	0.989	15	5	0.5–100
DMNB	4.6 (0.5)	194.1135	−0.52	50 ± 10	13	34.4	0.982	0.993	350	740	1–100
2,3-DNT	5.4 (0.7)	182.0333	−0.55	94 ± 7	23	42.4	0.995	0.989	715	7	0.5–50
NG	5.3 (0.5)	61.9884	+1.61	80 ± 15	23	19.2	0.999	0.999	20	18500	25–100
TNT	5.1 (0.4)	227.0184	−1.32	10 ± 7	25	46.5	0.994	0.979	25	10	2.5–50
2,6-DNT	5.8 (0.4)	182.0333	−0.55	91 ± 7	6	19.8	0.993	0.999	135	10	0.5–100
DADP	5.3 (0.5)	89.0597	−4.49	50 ± 10	26	14.9	0.999	0.999	10	370	0.5–100
2,4-DNT	6.0 (0.7)	181.0255	−0.55	79 ± 13	24	44.4	0.997	0.996	10	9	0.5–100
TATP	6.4 (0.5)	89.0597	−3.37	43 ± 5	5	27.2	0.998	0.982	105	89	0.5–100
2-NT	6.9 (0.7)	136.0404	0	72 ± 4	26	10.0	0.998	0.924	1090	3700	5–100
4-NT	7.2 (0.5)	136.0404	0	50 ± 10	27	10.7	0.999	0.995	255	1850	2.5–100
3-NT	—	137.0482	0	70 ± 13	22	n.d.	0.999	n.d.	82660	n.d.	n.d.

n.d. = not detected; ^a^n = 6 for 0.135 dm^3^ voids spiked with 5 µg explosives; samplers exposed for 120 h and samples injected in triplicate (n = 18); ^b^n = 6 for 0.135 dm^3^ voids spiked with 5 µg explosives; samplers exposed for 72 h and samples injected in triplicate (n = 18); ^c^n = 16 concentrations were prepared in duplicate (0.001–100 µg mL^−1^) and injected in triplicate (n = 96); ^d^0.135 dm^3^ voids spiked at five exposure levels i.e. 0.05–10 µg (n = 3 for each, n = 6 at 5 µg), samplers exposed for 120 h and samples injected in triplicate (n = 54).

When considering alternative detection devices for explosive vapours, there are very few that are comparable in terms of sensitivity, analyte diversity, portability, low cost and simplicity/ease of use. More recently, there has been a number of active samplers developed for the detection of explosive vapours such as electronic noses^22,23^, colorimetric and fluorometric sensor arrays also known as ‘optoelectronic noses’^24,25^, nanosensors^26^ and more recently polymer coated electrochemical and electromechanical sensors^20,27^. Although a number of these devices have been applied for multi-analyte detection such as the colorimetric optoelectronic nose sensor developed by Askim *et al*. (2016), most of the devices which show comparable or greater sensitivities to this passive sampling device have been developed for single analyte or small analyte group detection. One of these devices is a fluorescent probe for peroxide explosive vapours using an aromatic (multi-formyl phenol) aldehyde (diethylamine) oxidation reaction^28^. Hydrogen peroxide, DADP and TATP vapours were detected and a detection limit of 0.2 µg dm^−3^ for TATP vapour in 100 seconds. This system has only been applied thus far to peroxide vapours in controlled experimental conditions where it is assumed vapours have been allowed time to equilibrate in the measured void. The passive sampling device developed here can also detect TATP and DADP vapours, but it has a reproducible detection limit of 0.089 µg dm^−3^ for TATP which makes it at least twice as sensitive. As previously mentioned, a polymer-coated micromechanical cantilever-based sensor was developed for the selective pre-concentration of 2,4-DNT vapours^20^. Sensitivity was measured at mid µg dm^−3^ level, however, following further optimisation of the gas flow and vapour release rates, the authors proposed detection limits at sub-ng dm^−3^ levels could be yielded which would measure slightly lower than this passive sampler, however, again, this device was selective for 2,4-DNT vapours only. Silicon nanowire arrays have provided the highest sensitivities for TNT detection with detection limits measured at 0.01 ng dm^−3^ levels with further analyte differentiation work proposed to increase the selectivity of nitro-containing explosives^26^. Similar sensitivities have also been demonstrated from a dynamic planar solid phase microextraction (PSPME)-IMS detector with limits of detection for TNT and 2,4-DNT vapours measuring at 0.6 ng dm^−3^ ^14^. Recently, Zamora and colleagues studied active sampling of low to sub ng dm^−3^ levels of four explosives using fibreglass/stainless steel sampler substrates coated in PPPO-GR^29^. Analysis was performed using TD-MS incorporating a differential mobility analyser (DMA) and a peak discriminating algorithm to aid with interference reduction. Atmospheric background interferences did exist which limited detection at the pg dm^−3^ levels for PETN and NG. However, background interference was reduced sufficiently to enable excellent detection limits for RDX and TNT at the 0.01 ng dm^−3^ concentration level. Based on current literature therefore, the passive sampler developed herein, when combined with LC-HRMS analysis, is one of the most sensitive, broadly applicable, flexible and discriminating vapour detection methods available and has the added potential for tentative identification of new explosives components in post-analysis datasets^30^.

### Passive sampler materials optimisation

#### Sampler sorbent selection

A total of six sorbents were assessed for performance including Tenax TA (PPPO), Tenax GR (PPPO-GR), polydimethylphenyleneoxide (PPO), 1,3-diphenoxybenzene (1,3-DPB), triethanolamine (TEA) and polydimethylsiloxane (PDMS). Each of these sorbents was coated on a Nomex substrate and exposed to the analyte mix over a 72-h passive analyte uptake experiment. Nomex is a thermally resistant aromatic polyamide widely used for swabbing in aviation security and was selected as the sampler substrate for initial testing. Sorbent performance was assessed based on analyte recoveries and the results are depicted in Figure S1. As shown, PPPO-based sorbents achieved the highest recoveries for most analytes. Due to a non-parametric dataset, a Mann-Whitney U test (t-test for non-parametric data) was performed across sorbents to determine significant differences in analyte recoveries. Significant differences between sorbent uptake efficiencies were highlighted for HMTD (between PPO and 1,3-DPB, though recoveries were generally low anyway), DADP (between PPPO-based sorbents and all others except PPO), and TATP (between PPPO-GR and PDMS/1,3-DPB, and PPO and PDMS). Though no overall statistical differences existed between the two best sorbents (PPPO and PPPO-GR) in terms of analyte recovery in general, PPPO was selected for all further experiments for three reasons: (a) PPPO yielded lower variance in recovery for the majority of analytes than PPPO-GR, (b) PPPO performed better for TATP which is a current priority for security services to detect improvised explosives and (c) PPPO-GR is a stronger trap for other organics and blank sampler extracts of this material displayed more complex spectra when analysed by LC-HRMS. This could potentially interfere with low-level analytical measurement, especially when using lower resolution in-service explosives detection equipment. Further optimisation of film chemistry compositions for all sorbents was not performed. However, it is possible that changing such compositions or combining chemistries together could enhance sampler performance even further, but this was beyond the scope of this study. Following this, an initial assessment of recovery using a range of PPPO solution concentrations (20, 40 and 80 µg mL^−1^) coated on Nomex showed that a 40 µg mL^−1^ PPPO coating solution performed best in terms of analyte recovery and the solution viscosity produced an even coating on sampler.

#### Passive sampler substrate material selection

**Figure 1:**
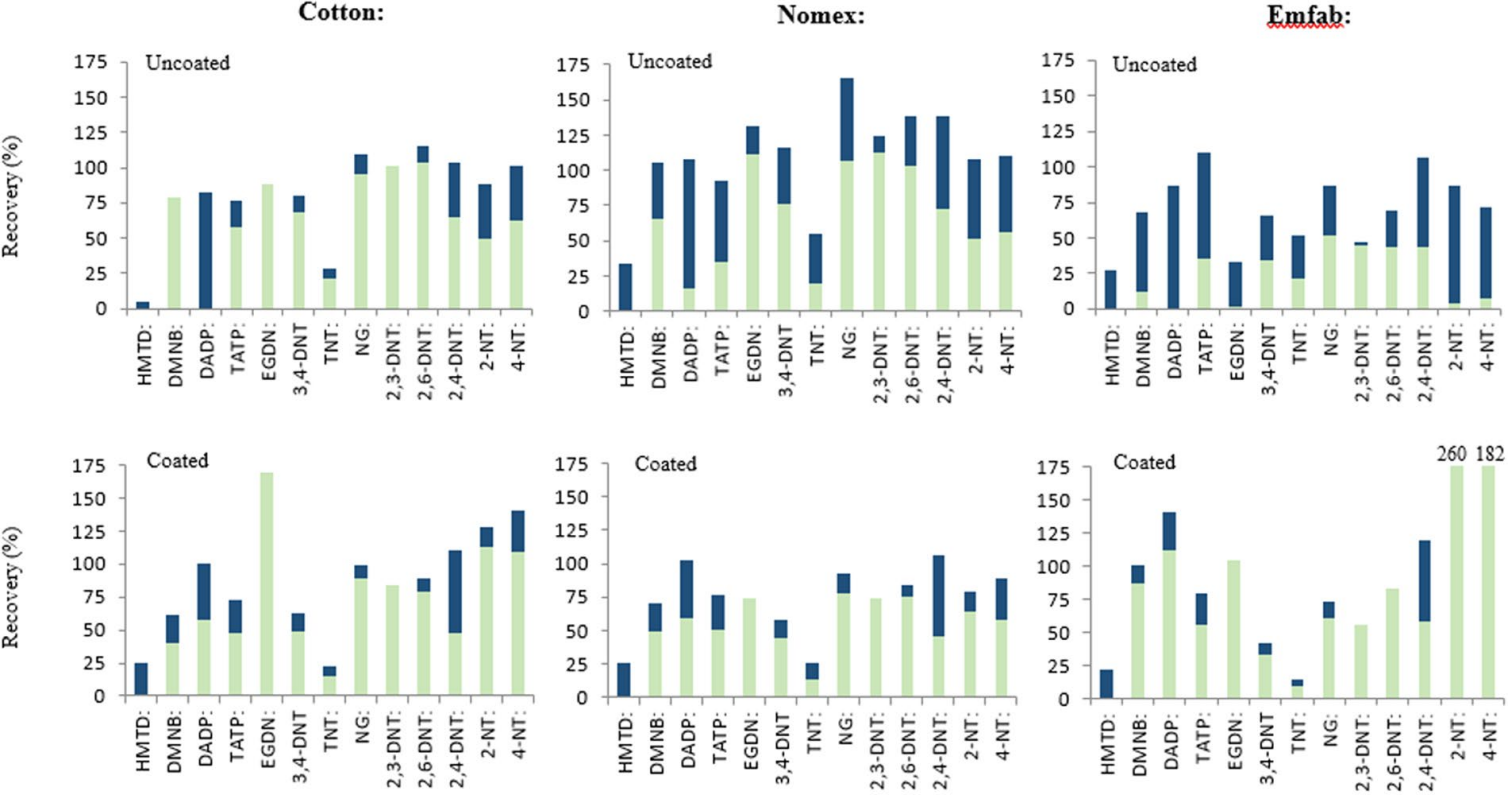
Analyte recoveries determined from via passive uptake over 72 h (green bars only) as a proportion of their recoveries determined from direct spiking of standards onto coated and uncoated Nomex, cotton and Emfab substrates (green and blue bars together).

Thermally stable substrates including cotton, Teflon, Teflon-coated fibreglass (Emfab) and Nomex were compared. Following a 72-h passive uptake exposure period, the adsorbent performance of all four substrates was assessed when each used as (a) a bare substrate (no sorbent coating) and (b) when each was coated in PPPO. As solvent extraction may not be as efficient as thermal desorption, this comparison of performance included correction of recoveries by spiking analytes directly on to PPPO-coated and bare Nomex, cotton, Teflon and Emfab substrates. Both coated and uncoated Teflon yielded <1% analyte recovery and was not considered further. Analyte recoveries on bare and PPPO-film coated Nomex, cotton and Emfab substrates are shown in Fig. 1 with the green bars representing passive uptake recoveries and combined blue and green bars representing recoveries following direct spiking of the sampler. As expected, analyte recoveries achieved via direct spiking were mostly greater than those achieved via passive sampling, particularly for uncoated substrates. Recoveries for analytes spiked directly onto uncoated substrates were in most cases greater than those achieved for samplers with a PPPO-coating applied, which may be a result of analyte evaporation due to the longer drying times of the applied analyte solution on the coated sampler. Analyte recoveries <100% for directly spiked coated samplers highlighted solvent extraction inefficiencies caused by high retention strength of the PPPO-coating. However, for screening purposes, the intention here was for integration with thermal desorption sources instead and no evidence of carryover was observed in any part of the study from successive thermal treatments. In the cases of uncoated Nomex and coated cotton and Emfab, analyte recoveries of >100% were likely observed as a result of preparation of the matrix-matched standard. Every attempt to minimise errors was made, but sampler exposure while spiking solutions were drying to external factors such as temperature and humidity, may have affected the recovery measurement through evaporation of analytes from the surface. Furthermore, Newsome *et al*. showed that small changes in humidity around the ion source of an APCI mass spectrometer can cause significant variations in the mass spectral response for HMTD and this may also be the case for other explosive molecules forming protonated and radical ion forms^31^. Overall, a significantly higher amount of peroxide explosives e.g. DADP and TATP, were retained on coated samplers.

**Figure 2:**
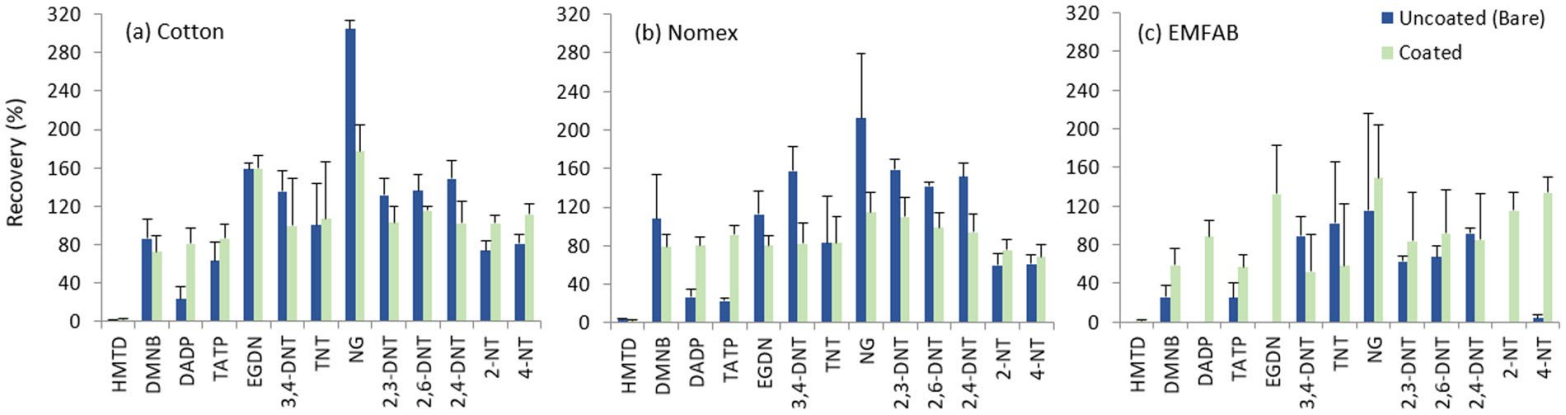
Comparison of analyte recoveries on bare and PPPO-film coated cotton (**a**) Nomex (**b**) and Emfab (**c**). Substrates were analysed following removal from the source after a 72-h exposure and a 7-day depuration period (n = 3).

A 7-day retention study was also performed on uncoated and PPPO-coated Nomex, cotton and Emfab substrates. As shown in Fig. 2, analytes were retained for longer on the coated samplers once removed from the source. As the coating of the sampler may vary from sampler to sampler, it is important that the substrate also acts as an efficient trap for explosive components in the absence of the source-equilibrated environment. For this reason, the Emfab substrate was not considered for further testing as passive uptake recoveries on the uncoated sampler were shown to be significantly less than recoveries on the coated sampler and almost half of the analytes were not retained on sampler following the 7-day retention study. In particular, NG and 4-NT on Emfab was only detected in two of the three exposed samplers (Fig. 2). This was also the case for HMTD on bare cotton and Nomex substrates, and for EGDN and 2-NT on bare cotton. Therefore, uncoated substrates in general did not show stable retentivity of explosives vapour following removal from the source. Coated Nomex and cotton were found to achieve the best performances overall. Further sampler substrate assessment and applications testing in various external environments using in-service explosive detection equipment was carried out to assess the suitability of coated Nomex and cotton samplers for this application and will be described later in this paper.

### Passive sampling kinetics and analyte recovery

**Figure 3:**
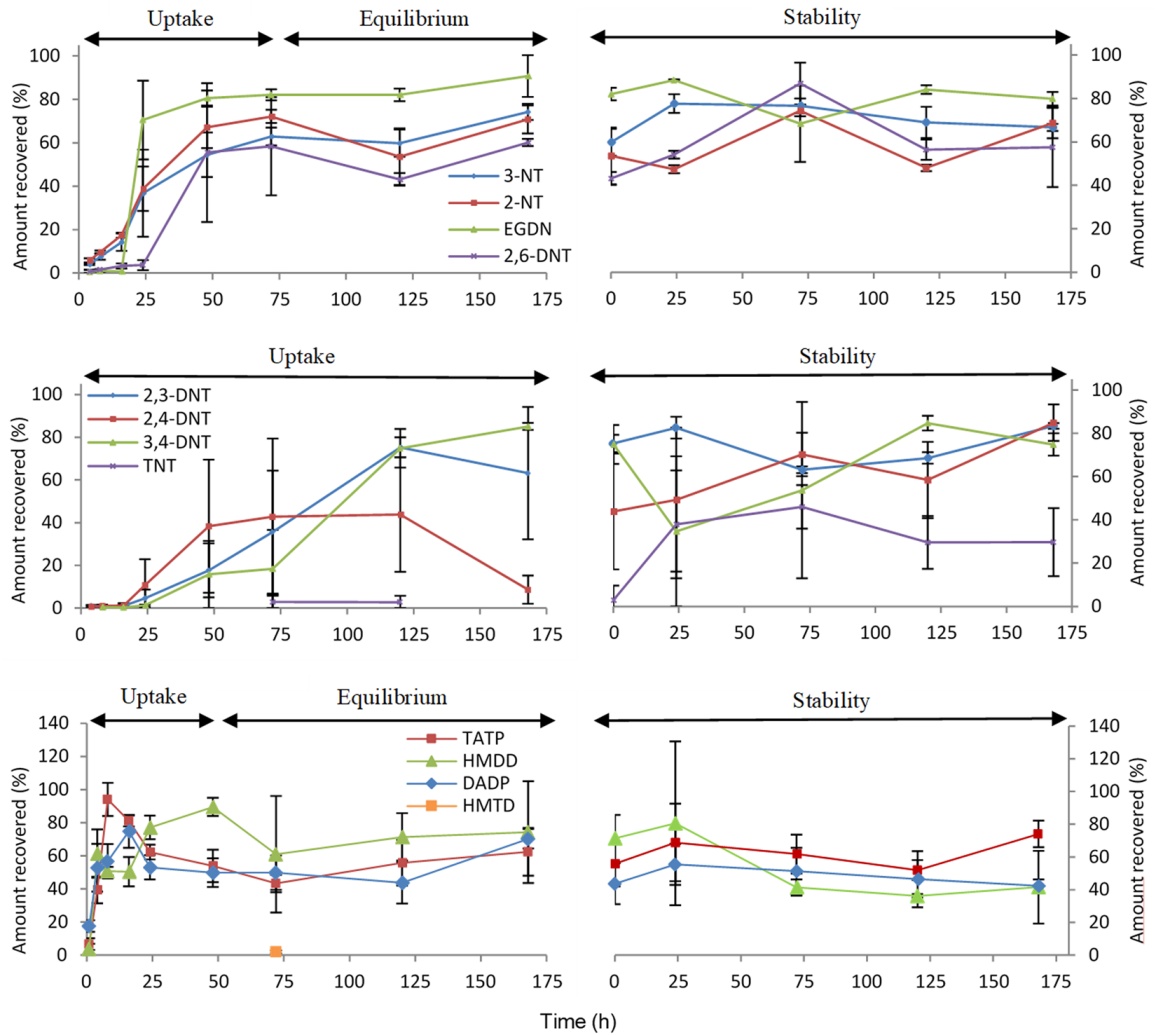
Passive uptake (168 h) and retention studies (168 h) for 12 explosive analytes. Retention studies commenced following exposure to source and removal of sampler to clean vessel (1 L). Error bars show the standard deviation between samples (n = 3).

Duran glass bottles were considered the most practical means to enable a large quantity of parallel experiments to be performed using a standardised volume (0.135 dm^3^). Although this set-up minimised the possibility for cross-contamination or interference (in comparison to room-scale exposures) and allowed for inter-comparison studies to be performed for different sorbent and substrate combinations, it was acknowledged that the distance between source and sampler was reduced significantly in comparison to a more realistic environment. In addition, the sampler was inserted at the same time as the explosive trace and the concentration of explosive in the void did not reach equilibration at time zero. However, this approach was necessary to minimise the loss of vapours from the glass bottle before the cap was fitted. Despite its limitations, this small-scale arrangement was considered a reasonable means to demonstrate sampler proof-of-concept early in the development process and uptake kinetics were tested again more comprehensively in Part B in more realistic void environments at significantly larger scales. Arguably, this also potentially represents a more realistic assessment than voids which are pre-equilibrated with analyte vapour before sampling occurs. Normally, there are three phases of uptake of an equilibrated gas at a fixed concentration onto a surface, i.e. a linear uptake phase, followed by a curvilinear stage, and then equilibrium where the concentration on the adsorbent surface remains constant^32^. In the situation where an explosive device is deployed, there will also be a lag period prior to analyte uptake. In this scenario, the distance of the sampler to the explosive source would be unknown and if the passive samplers are used routinely, the source would most likely be placed in the area before or after the sampler is deployed. Analyte recovery, uptake and retention studies on sampler were assessed for 12 selected analytes (shown in Fig. 3) due to standard availability, coverage of explosive classes and likelihood of uptake based on vapour pressure (analytes with lower vapour pressures were preferred as any observed uptake would signal potential uptake of same-class analytes with higher vapour pressures).

**Figure 4:**
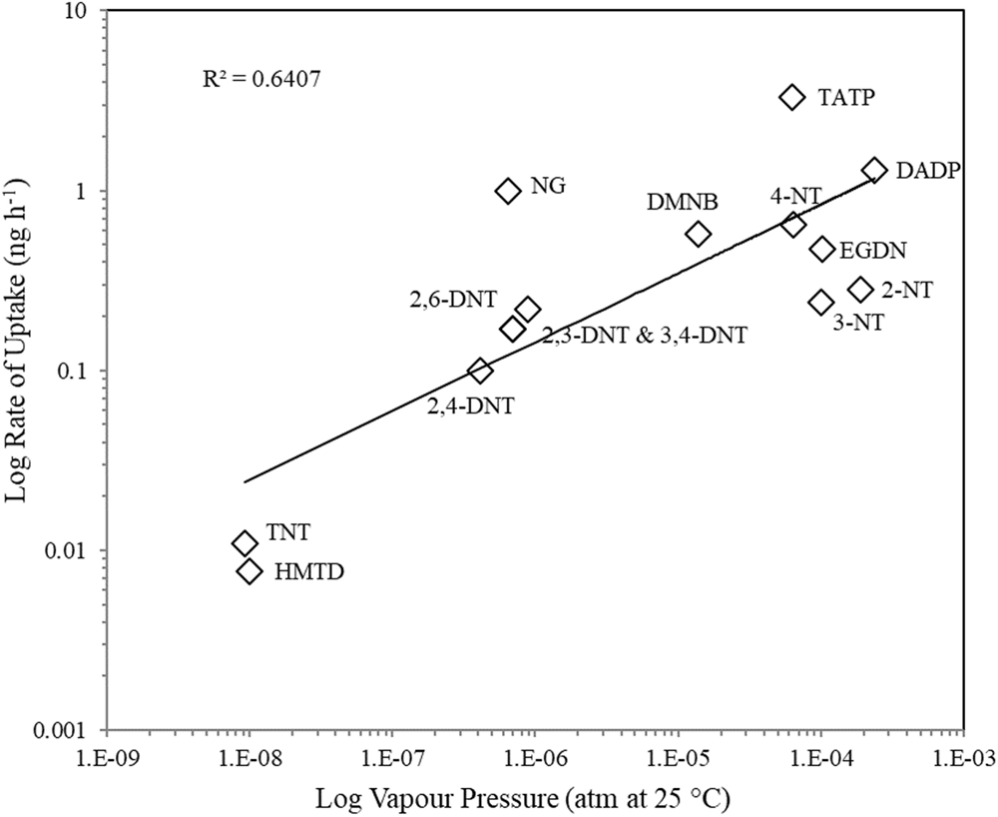
Correlation between rate of uptake on sampler (ng h^−1^) and vapour pressure (atm at 25 °C) for each analyte.

Average analyte recoveries were measured at ~70% and mostly ranged between 40–95% once equilibrium was achieved. Uptake for most compounds reached a maximum or plateau between 3–5 days, and all analytes, except TNT, HMTD and 3,4-DNT, were detected and quantified on sampler within 4 h of deployment. TATP, DADP and HMDD had the shortest lag period and were detected on sampler as soon as 1 h after exposure (this earlier time point was included for peroxide studies only). Given their lower vapour pressures, it was not surprising TNT and HMTD were only detected at or after 72 h, measuring at approximately 2%. It is advised that a 10^−9^ atm vapour pressure (at 25 °C) be used as a minimum cut-off for target analyte selection using this sampling device. Furthermore, stability of HMTD in the gas phase under ambient conditions is limited and its detection at all after such a time-period was unexpected^31,33^. Variance in measured compound recoveries across several time points was observed particularly for DNT isomers, but this did not affect detection in any case beyond the 24 h time point. All other compounds showed repeatable kinetics measurements and good stability on sampler. Retention studies involved removal of the exposure source after 120 h (average time of plateau for analytes) to a clean Duran bottle and analysis of the sampler after 24, 72, 120 and 168 h. As shown, most analytes were retained on sampler for a period of up to 7 days after exposure, with low variance measured between replicate experiments. Only HMTD was the exception to this and could be partly explained by its very low uptake during the exposure period. Figure 4 highlights the correlation between vapour pressure and uptake on sampler, where the rate of analyte uptake at room temperature (21 °C) was calculated based on the time taken to complete the linear uptake phase (shown in Fig. 3).

Regarding sampler stability and storage over longer periods, high analyte recoveries were achieved over a 22-month testing period and no statistical differences were found between sample ages and storage conditions (see Figure S2). Therefore, sampler recovery was shown to be unaffected over time and it is recommended that samplers be stored in a tin (or a sealed, opaque container) at room temperature in air or under vacuum for a period of up to 22 months.

### Interference assessment of passive samplers

**Figure 5:**
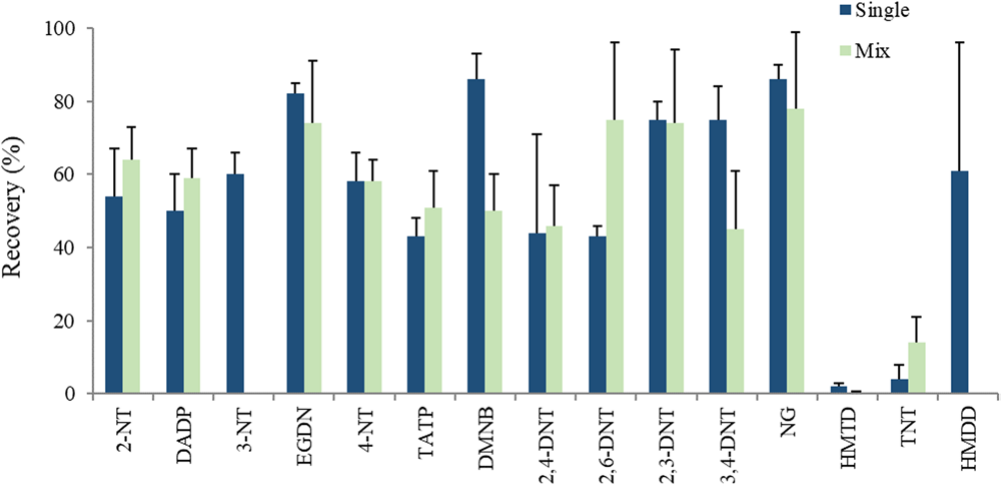
Recoveries (n = 6) for each explosive vapour on sampler when exposed over 72 h individually and as a mixture.

Competitive uptake of co-exposed explosive vapours was examined first (samplers were exposed to 5 µg of each analyte together over 72 h in 0.135 dm^3^ flasks). Compared to uptake of analytes exposed individually, the uptake for most analytes when exposed as a mixture differed <15%, as shown in Fig. 5. Both HMTD and HMDD showed little or no uptake when exposed as a mixture. Low uptake of HMTD may be explained by its mid-low vapour pressure and for HMDD, its low stability. During these experiments, HMDD was withdrawn from sale by the manufacturer and was not used in any further experiments. In the mixture, the recovery of TNT doubled and, interestingly, 2,6-DNT and 3,4-DNT increased and decreased in concentration, respectively, by almost equal amounts. There does not seem to be a clear reason as to why or how 3,4-DNT may have converted to the 2,6-DNT isomer, however, the latter seemed to be relatively more stable. 3-NT was not detected at low level concentrations using the developed LC-HRMS method, therefore, sampler recovery of 3-NT when exposed as a mixture was not determined.

Where samplers are intended for deployment in operational environments, it is important to acknowledge that other compounds may exist that may mask detection. The effect of non-explosives related interferences arising from a range of simulated operational environments on sampler performance was assessed next using both LC-HRMS and in-service TD-MS and IMS instrumentation employed by law enforcement agencies for in-the-field analysis. The use of thermal desorption as the sample introduction technique for such in-service instrumentation enabled direct analysis of samplers without the need for extraction. This represents a very convenient approach for fast field-based analysis and was considered suitable for integration with high intensity and high frequency searches (only a few seconds were required for each measurement). However, these techniques are usually only sufficient for qualitative screening. Though slower and less practical for field measurements, the advantage of using an optimised LC-HRMS method is that it can be used quantitatively and can utilise both retention time and full-scan high resolution accurate mass measurement to tentatively identify potentially new explosives threats with much higher assurance without the need for reference standards in the first instance and retrospectively in accrued datasets. Using these techniques with a passive vapour sampler potentially improves their sensitivity and allows a larger area to be covered in a single measurement.

Samplers were exposed in an office, a commercial kitchen and an air-polluted central London roadside site for 7 days. No explosives were placed in any of these locations for safety reasons. However, these samplers were subsequently exposed to 5 µg of each analyte as a mixture over 5 days in 0.135 dm^3^ flasks in the laboratory to determine whether their detection was impeded as a result of co-adsorbed interferences from each environment. For this section of work, coated Nomex and cotton substrates were examined, as they showed similar performances for analyte uptake up to this point. Following a 7-day exposure and removal of samplers from all environments, there was a noticeable change in colour for the samplers exposed at a roadside site (light brown) and in a kitchen (faint yellow). No colour change was observed for those exposed in an office environment. Sampler interference was assessed and although signal suppression was observed using liquid extraction and LC-HRMS analysis at ~30% in the office, 50% in the kitchen and 50–70% at the roadside compared to freshly made samplers, detection of most analytes using currently in-service detection equipment was still possible (Figures S3 and S4 for IMS and TD-MS analysis results, respectively). Analysis of one sampler for the presence of EGDN exposed to office interferences yielded a false negative using IMS. More replicates would have enabled a fuller characterisation of detection frequency to be determined. Furthermore, and although field instruments such as the IMS used in this study do undergo rigorous testing to ensure sufficient quantitative response for security application, detection frequency could drop for field systems at lower concentration levels. Detection frequency is shown in more detail in Part B of this work and across a range of more operationally relevant scenarios. That said, in this preliminary assessment, Nomex substrates worked well in particular and showed lower signal variability for all compounds using the in-service field equipment. However, using LC-HRMS, a few instances occurred where a single false negative result was recorded within the three replicate measurements for (a) EGDN on Nomex using IMS analysis (this is most likely due to some instability at desorption temperatures much greater than its boiling point), and (b) TATP and DMNB on cotton using LC-HRMS (Figure S5). As the signal for both TATP and DMNB should have been detected by HRMS at the spiked analyte concentrations, this false negative result, as well as the lower signal suppression observed for most analytes using IMS and TDMS analysis, were the main reasons for selecting Nomex as the sampler substrate for all further experiments. Though signals for HMTD were statistically improved in the kitchen environment (Figure S5), recovery was still <1% and variance remained high. All other exposed analytes were detected in the three replicate samples using PPPO-coated Nomex.

The third assessment of interferences focussed on exposure of the sampler to a selection of commonly used personal care product and household cleaning agent vapours and were also co-exposed to analyte vapours at the same time to assess any impediment to their detection. As these were confined within a relatively small volume void and present at unrealistically high concentrations, this arguably represents a worst-case scenario for potential interference from such compounds. Using IMS analysis, the signal for EGDN varied greatly again, and in most cases, was only detected on one of three exposed samplers, including samplers exposed to the analyte mix only, and was not detected at all when co-exposed with bleach. TNT channels were suppressed in the presence of all interferences and was only detected on one of the three samplers when co-exposed with multi-surface spray. Interestingly, TATP was present in the analyte mix exposed to the sampler and was only detected from a sampler co-exposed with bleach, highlighting analyte enhancement in the presence of this interferent as TATP was not detected on samplers exposed to bleach only. Lastly, although one HMTD IMS channel alarmed when analysing three samplers co-exposed with bleach, detection was not confirmed with the second HMTD channel.

When using TD-MS, most analytes monitored in negative mode (i.e. NG, EGDN and DNT) were detected for n = 2 except for TNT which was not detected on samplers co-exposed with bleach and for one out of two samplers co-exposed with aftershave. In positive mode, TATP was only detected when co-exposed with multi-surface spray and hairspray using TD-MS analysis. Samplers exposed to targeted interferences only were analysed in positive and negative modes and no false positive results were recorded. Relative to samplers exposed to the analyte mix only, all LC-HRMS analyte signals were enhanced when the sampler was co-exposed with multi-surface spray and in the cases of EGDN and all NT isomers, signals were enhanced for all co-exposed samplers (Figure S6). Bleach and aftershave were found to cause the most signal suppression of analyte vapours overall. HMTD was not detected in this experiment which corresponds to the results produced with IMS and TD-MS analysis. NG was only detected on one of three samplers co-exposed with bleach and not at all when co-exposed with aftershave. However, this may be due to the poor sensitivity for NG using LC-HRMS analysis as its detection rate was not affected when using IMS and TD-MS analysis.

## Conclusions

The performance of the developed passive sampling device is very promising, both under laboratory controlled conditions and in simulated operational environments. The sampler was evaluated for 15 explosive components of mid-high vapour pressures (cut-off at ~10^−9^ atm at 25 °C) and the optimum exposure time was determined between 3–5 days with uptake as early as 1–4 h and retention of analytes on sampler for up to 7 days following removal from source. The developed passive sampler is readily integrated with in-service instrumentation such as IMS and TDMS, and has successfully detected explosive vapours from commercial explosive standards when exposed over time. Sampler interference was assessed and although signal suppression was observed, this did not affect the detection frequency of most analytes on currently in-service detection equipment. In the case of EGDN and TNT, there was some instances where a false negative was reported for trace amounts on coated Nomex pre- or co-exposed which is likely due to lower instrument sensitivity for EGDN on the IMS and low uptake of TNT (lowest vapour pressure) on sampler. It is recommended that at least two samplers are deployed so that analysis can be carried out in duplicate or using complementary technologies to minimise the occurrence of false negative results. Following the successful demonstration of sampler feasibility in open environments, the developed liquid chromatography-high resolution mass spectrometry (LC-HRMS) method as well as the currently in-service spectrometric methods were further applied to semi-operational and realistic environments and venues (presented in Part B) to determine the most effective placement of the device in a room, suitability of sampler casing prototypes, false positive/negative rates and alternative sampler applications.

## Methods

### Reagents and materials

High performance liquid chromatography (HPLC) grade methanol and acetonitrile and analytical grade dichloromethane were purchased from Fisher Scientific (Loughborough, UK). Standard solutions of 2,4-dinitrotoluene (2,4-DNT, 1000 μg mL^−1^ of >99% purity), 2,6-dinitrotoluene (2,6-DNT, 1000 μg mL^−1^ of >99% purity), 3,4-dinitrotoluene (3,4-DNT, 1000 μg mL^−1^ of >99% purity), trinitrotoluene (TNT, 1000 μg mL^−1^ of >99% purity), nitroglycerin (NG, 1000 μg mL^−1^ of >99% purity), 2-nitrotoluene (2-NT, 1000 μg mL^−1^ of >99% purity), 3-nitrotoluene (3-NT, 1000 μg mL^−1^ of >99% purity), 4-nitrotoluene (4-NT, 1000 μg mL^−1^ of >99% purity), triacetone triperoxide (TATP, 100 μg mL^−1^ of >99% purity), hexamethylene diperoxide diamine (HMDD, 100 μg mL^−1^ of >99% purity), hexamethylene triperoxide diamine (HMTD, 100 μg mL^−1^ of >99% purity) were purchased from Kinesis Ltd. (St. Neots, UK). Ethylene glycol dinitrate (EGDN, 1000 μg mL^−1^ of >98% purity) was sourced from Thames Restek Ltd. (High Wycombe, UK). A solution of diacetone diperoxide (DADP, 10000 μg mL^−1^) in acetonitrile was obtained from the Forensic Explosives Laboratory (Dstl, Fort Halstead, Kent, UK). Solid standards of 2,3-dinitrotoluene (2,3-DNT, >98% purity), 2,4-DNT (>97% purity) and 2,3-dimethyl-2,3-dinitrobutane (DMNB, >98% purity) were obtained from Sigma-Aldrich (Gillingham, UK) and stock solutions of 1000 μg mL^−1^ were prepared in methanol. Working solutions (100 μg mL^−1^) of each 1000 μg mL^−1^ stock solution were also made up in HPLC grade methanol. All stock and working solutions were stored in the dark at 4 °C, except for EGDN and HMDD which were stored at −20 °C. Ammonium acetate (>99% purity), ammonium chloride (>99% purity) and hydrochloric acid solution (37% *w/v*) were obtained from Sigma-Aldrich. Ultra-pure water (18.2 MΩ cm) was delivered from a Millipore Milli-Q water ultra-purification system (Millipore, Bedford, MA, USA). All commercially available reagents were used without further purification.

Sampler substrate materials tested included Nomex (DSA Detection, MA, USA), cotton (Smiths Detection, UK), Teflon (Savillex, MN, USA) and Teflon-coated glass fibres (*Emfab*, Pall, UK). For sampler sorbent films, Tenax TA (PPPO, 60–80 mesh, Sigma-Aldrich), Tenax GR (PPPO-GR, 60–80 mesh, Thames Restek), poly (2,6-dimethyl-1,4-phenylene oxide) (PPO, Sigma-Aldrich), 1,3-diphenoxybenzene (1,3-DPB, Santa Cruz Biotechnology, TX, USA), triethanolamine (TEA, ≥99% purity, Sigma-Aldrich) and polydimethylsiloxane (PDMS, viscosity 200 cSt, Sigma-Aldrich) were used.

### Preparation of passive samplers and liquid extraction procedures

Passive samplers were prepared by coating swabs in a 40 mg mL^−1^ (PPPO, PPPO-GR, PPO and 1,3-DPB) or 4% (*w/v*) (TEA and PDMS) solution in dichloromethane and allowed to air dry. The optimised passive sampler was composed of a Nomex substrate coated in a PPPO film and these were used for all subsequent recovery and application studies in Parts A and B of this work. Prior to exposure, samplers were washed twice in methanol to remove contaminants. Following exposure, samplers for either LC-UV or LC-HRMS analysis, were placed in a screw capped, septum lined glass vial (7 mL) and extracted in 0.5 mL methanol. Aliquots of these extracts were placed into 250 µL glass inserts (Agilent, UK) within LC vials, and were crimp-capped and stored at −20 °C until analysis.

### Sampler recoveries, passive uptake kinetics and analyte retention

For measurement of passive uptake and determination of subsequent recovery from the sampler, 5 µg or 15 µg of individual standards were deposited at the bottom of 0.135 dm^3^ Duran glass bottles. These quantities reflected a realistic explosive mass to void ratio (i.e. ≤~111 µg per dm^3^) and which were consistent with other works on trace detection of explosives^34^ and with quasi-realistic applications simulated in Part B (e.g. equivalent of ~4.3 g of Perunit 28E in a 38,500 dm^3^ ISO shipping container). Passive samplers were secured in the lid of the container and sealed (~115 mm in distance from spiked standard solution). The uptake kinetics for all analyte vapours were characterised from 1 hour up to 7 days (n ≥ 5 time points, all exposed in triplicate). Analyte retention studies involved the passive uptake of the individual analytes over 5 days and transfer of the exposed sampler to a clean, explosives free and larger void (1.35 dm^3^). Studies of sampler losses to this void were also carried out in triplicate over four timepoints between 24 hours and 7 days. Exposed samplers were extracted and analysed by LC-HRMS (peroxides only) or LC-UV (remaining compounds) and compared to a standard. For direct spiking of samplers, a mass of 5 µg of each analyte was directly spotted on to the sampler using 50 μL of 100 μg mL^−1^ standard solution and allowed to dry. All experiments were performed under controlled temperature (21–23 °C) in triplicate and included negative controls. For long term stability (i.e. shelf-life), samplers were stored under vacuum and in a metal container at ambient conditions for 22 months. Recovery was retested at 1, 3, 6, 12 and 22 months using EGDN, 2,3-DNT, 3-NT (selected to cover high-mid vapour pressure ranges). Passive uptake experiments (72 h) were carried out in triplicate and aged samplers from both storage media were compared to freshly prepared samplers.

### The effect of background interference on sampler performance

For the competitive uptake study, samplers were exposed to the selected analyte vapours (5 µg each) both individually and as a mix over 72 h in 0.135 dm^3^ flasks as above. For the assessment of interferences from different open environments (no explosives present), samplers were exposed in an office, a commercial kitchen and an air polluted central London roadside site for up to 7 days. Samplers were subsequently removed and then exposed as above for 5 days to a mixture of explosive vapours spiked at 5 µg in the 0.135 dm^3^ Duran (TNT, 2,4-DNT, EGDN, and NG for IMS and TD-MS analysis and all analytes for LC-HRMS analysis). Sampler performance was also tested in the same way using a selection of potentially interfering compounds with high vapour pressures. Samplers were co-exposed for 5 days to a selection of explosives (TNT, 2,4-DNT, EGDN, NG, TATP and HMTD for IMS and TD-MS analysis and all analytes for LC-HRMS analysis) along with commonly used household and personal care products including multi-surface spray, hairspray, bleach and aftershave (250 µL of product was placed in an open glass vial within the void to prevent mixing with the analyte solutions prior to uptake). In each case, at least three replicate exposures were performed for measurement with each analytical technique. For IMS and TD-MS analysis, two samplers were analysed in negative mode and one sampler was analysed in positive mode.

### Instrumentation, analytical methods and conditions

For analytes bearing a UV chromophore (i.e. all except peroxides), the quantitative analytical method for recovery assessment, kinetics and retention experiments employed an Agilent HP 1100 series liquid chromatograph (Agilent Technologies, Berkshire, UK) and a Waters Sunfire C_18_ column (2.1 × 150 mm, 3.5 μm, Waters UK, Milford, MA, USA). The isocratic mobile phase consisted of 60:40 (*v/v*) 8 mM ammonium acetate in methanol:water at a flow rate of 0.15 mL min^−1^ and an injection volume of 2 μL. The column temperature was 45 °C. Diode array detection (DAD) was performed at 210 nm. This method was only required for single compound exposures and, as such, baseline separation of all analytes was not optimised under these conditions.

Confirmatory analysis for all 15 explosives was performed using liquid chromatography coupled to high resolution accurate mass spectrometry (LC-HRMS). Sample injection (5 µL) was performed using a Thermo HTS-A5 autosampler with sample storage compartment temperature control set at 10 °C. Separations were performed on a Waters Sunfire C_18_ column (2.1 × 150 mm, 3.5 μm) at a temperature of 44 °C. Mobile phases were 90:10 (*v/v*) 0.2 mM ammonium chloride in water:methanol (A) and 10:90 (*v/v*) 0.2 mM ammonium chloride in water:methanol (B). Separate methods for negative and positive ionisation modes were employed to maximise peak intensity and definition. For negative mode, an isocratic separation method was carried out using a mobile phase of 60% B with a flow rate of 0.2 mL min^−1^ and a total run time of 10 min. For positive mode, a gradient profile was carried out over 12 min at a flow rate of 0.3 mL min^−1^. Mobile phase was set at 20% B at 0 min and was raised to 100% B over 4 min and then held at 100% B for a further 2 minutes. Re-equilibration time was 5 minutes.

A Thermo Exactive instrument equipped with a heated atmospheric pressure chemical ionisation (APCI) source was used for HRMS. Nitrogen was used as a nebulising and desolvation gas within the ionisation source and the collision cell. High resolution mode was employed at 50,000 FWHM to allow sufficient chromatographic peak definition for quantitative measurements. Other HRMS conditions are given in the supplementary information (Table S1). For direct infusion, a Cole-Parmer 74900 Series syringe pump was set to deliver 10 μL min^−1^ of analyte solution from a 250 μL glass syringe. The scan range was m/z 50–400 for all MS experiments. All acquisition data was processed using Xcalibur v 2.0 software (Thermo). Mass spectrometric analysis was carried out in total ion scanning mode with targeted ions (shown in Table 2) extracted post-acquisition.

For applications testing, direct analysis of the passive sampler was performed using IMS (IONSCAN 500DT, Smiths Detection, Hemel Hempstead, UK) and a vehicle-mounted TD-MS instrument provided by DSA Detection (DSA Detection, North Andover, MA, USA) incorporating a SCENTINEL API 2000 Mobile Mass Spectrometer (AB SCIEX, Warrington, UK). Desorption temperatures were set at 205 °C and 190 °C for each instrument, respectively.

### Instrumental and method performance for LC-UV and LC-HRMS analysis

For both LC-UV and LC-HRMS methods, precision for retention time, peak area and peak height were assessed using replicate injections of a 1 µg mL^−1^ standard (n ≥ 6) and for LC-HRMS, this utilised the most intense ion to within a 5 ppm mass accuracy threshold^35^. For linearity and range, coefficients of determination based on peak height were assessed using standard solutions between 0.05–100 µg mL^−1^ for LC-UV (n = 11) and 0.001–100 µg mL^−1^ (n = 16) for LC-HRMS. For LC-UV, the LOD and LOQ were calculated using the signal-to-noise ratio of three low-level standard concentrations each injected in triplicate (n = 9; 3:1 and 10:1 for LOD and LOQ, respectively). For LC-HRMS, LOD and LOQ were determined based on ICH harmonised tripartite guidelines, i.e. three and ten times the standard deviation of the response divided by the slope of the calibration curve^36^. All LODs were retested by re-injecting the analyte at the calculated LOD value.

Using LC-HRMS only, method performance (i.e. passive sampling, solvent extraction and analysis) was also evaluated with respect to linearity, range, precision, sensitivity, recovery and matrix effects. For this, passive uptake of analyte mixtures was carried out over a 5 day period. For retention time and peak height precision, samplers were exposed to 5 µg of each analyte mix (n = 6). For range and linearity, samplers were exposed to 0.05–10 µg of each analyte (N = 5). Method LODs were defined as the concentration (calculated weight spiked in a 0.135 dm^3^ void) which produced a HRMS signal ≥2000 counts (without background noise) based on the calibration curve produced in the linearity assessment or a HRMS signal measuring at least three times the height of the baseline noise (where noise existed). All samples were injected in triplicate in both positive and negative modes, unless otherwise specified.

## Electronic supplementary material

Supplementary Information
