# Supplementary material for: Sorbent Film-Coated Passive Samplers for Explosives Vapour Detection Part A: Materials Optimisation and Integration with Analytical Technologies
Source: Sci Rep. 2018 Apr 11;8:5815. doi: 10.1038/s41598-018-24244-y (PMC5895691; doi:10.1038/s41598-018-24244-y)
Supplement: Supplementary file 1 — Supplementary Information [file 41598_2018_24244_MOESM1_ESM.pdf]

ELECTRONIC SUPPLEMENTARY INFORMATION

SORBENT FILM-COATED PASSIVE SAMPLERS FOR EXPLOSIVES VAPOUR DETECTION PART A: MATERIALS OPTIMISATION AND INTEGRATION WITH ANALYTICAL TECHNOLOGIES

Gillian L. McEneff[a*], Bronagh Murphy[a], Tony Webb[b], Dan Wood[b], Rachel Irlam[a], Jim Mills[c], David Green[a], Leon P. Barron[a*]

[a]*King's Forensics, School of Population Health & Environmental Sciences, Faculty of Life Sciences & Medicine, King's College London, 150 Stamford Street, London, SE1 9NH, United Kingdom.*

[b]*Threat Mitigation Technologies, Metropolitan Police Service, 113 Grove Park, London, SE5 8LE, United Kingdom.*

[c]*Air Monitors Ltd., 2/3 Miller Court, Severn Drive, Tewkesbury, Gloucestershire, GL20 8DN, United Kingdom.*

Table of contents:

S1.0 Instrumental performance for LC-UV analysis S3

List of tables:

Table S1. HPLC-UV method performance data for eight explosive analytes S4
Table S2. Optimised atmospheric pressure chemical ionisation (APCI) and mass spectrometric conditions.. S5

List of figures:

Figure S1. Average recoveries (72 h passive uptake) for selected analytes using Nomex coated with Tenax (PPPO), Tenax-graphite (PPPO-GR), polydimethylphenyleneoxide (PPO), 1,3-diphenoxybenzene (1,3-DPB), triethanolamine (TEA) and polydimethylsiloxane (PDMS). Error bars show the standard deviation between n=6 replicate samples.. S6
Figure S2. Passive uptake recoveries for three explosive components on samplers stored over time in a tin and under vacuum and compared to those freshly prepared. Sampler stability was measured over a 22-month period (except for 3-NT which was measured up to 12 months). Bars represent the average of n=6 replicates. Whiskers represent the standard deviation. S7
Figure S3. Assessment of interferences to analyte uptake on samplers following exposure to open environments using IMS analysis (n=2 samplers per exposure).

Bars and whiskers represent the average and maximum/minimum value. The bar marked with * represents the only 1 positive result measured from the 2 samplers exposed for EGDN (cumulative amplitude for negative results below IMS ‘positive’ threshold not available to reliably calculate average for n=2 including the negative result). S8
Figure S4. Assessment of interferences to analyte uptake on samplers following exposure to several simulated environments using TD-MS analysis (n=2 samplers per exposure). Bars and whiskers represent the average and maximum/minimum values, respectively S9
Figure S5. Assessment of analyte uptake on Nomex and cotton samplers following exposure to open environments using LC-HRMS analysis (n=3 samplers per exposure) S10
Figure S6. Assessment of analyte uptake on Nomex sampler when co-exposed with targeted interferences using IMS analysis (bars represent average for n=3 ±standard deviation unless otherwise marked). Bars marked with * represent values for only n=1 sampler of the three deployed which yielded a positive result for EGDN. Bar marked with ** represents the average value for n=2 samplers of the three deployed which yielded positive results for TNT. Cumulative amplitude for negative results below IMS ‘positive’ threshold were not available to reliably calculate an average for all n=3 replicates including the negative result(s). S11

S1.0 Instrumental performance for LC-UV analysis

For LC-UV, mobile phase composition (methanol and water), flow rates (0.15, 0.2, 0.3, 0.4 mL min^{-1}), sample injection volume (5, 10, 15, 20 µL), HPLC columns (ACE C_{18}-Aromatic and Waters Sunfire C_{18}) and column temperature (19, 28, 34, 39, 44 °C) were optimised. A 10 mM ammonium acetate solution in a mixture of methanol and water was selected as a suitable mobile phase. When the optimised conditions were determined, the Waters Sunfire C_{18} column was selected over the ACE C_{18}-Ar column as it offered the best resolution between two closely eluting DNT compounds i.e. 3,4-DNT and 2,3-DNT. The HPLC separation method developed was performance tested on the eight UV chromophore-containing analytes listed in Table S4. The results yielded for linearity, range, LOD, LOQ and reproducibility were deemed satisfactory for characterisation of prototype performance over the ng-µg range of compound on-sampler.

Table S1. HPLC-UV method performance data for the eight selected explosive components.

Analyte	Average retention time (min) ± RSD (%) n=12	LOD (µg mL^{-1}) n=9	LOQ (µg mL^{-1}) n=9	Linearity (R^2) n≥8	Linear range (µg mL^{-1}) n≥8
EGDN	4.2 ± 0.2	0.10	0.34	0.999	0.34-100
3,4-DNT	5.8 ± 0.1	0.16	0.53	0.997	0.53-100
2,3-DNT	6.2 ± 0.3	0.12	0.40	0.999	0.40-100
TNT	6.4 ± 0.9	0.07	0.23	0.999	0.23-100
2,6-DNT	7.0 ± 0.1	0.11	0.38	0.997	0.38-100
2,4-DNT	7.2 ± 0.3	0.12	0.40	0.996	0.40-100
2-NT	8.3 ± 0.1	0.06	0.20	0.995	0.20-100
3-NT	9.2 ± 0.1	0.08	0.28	0.996	0.28-100

Table S2. Optimised atmospheric pressure chemical ionisation (APCI) and mass spectrometric conditions.

	Positive mode	Negative mode
Sheath gas flow rate:	60	60
Auxiliary gas flow rate:	5	5
Sweep gas flow rate:	0	0
Spray voltage (kV):	4	4
Vaporiser temperature (°C):	300	300
Capillary temperature (°C):	250	250
Capillary discharge voltage (kV):	5	25
Skimmer voltage (V):	18	-18
Tube lens voltage (V):	50	-50

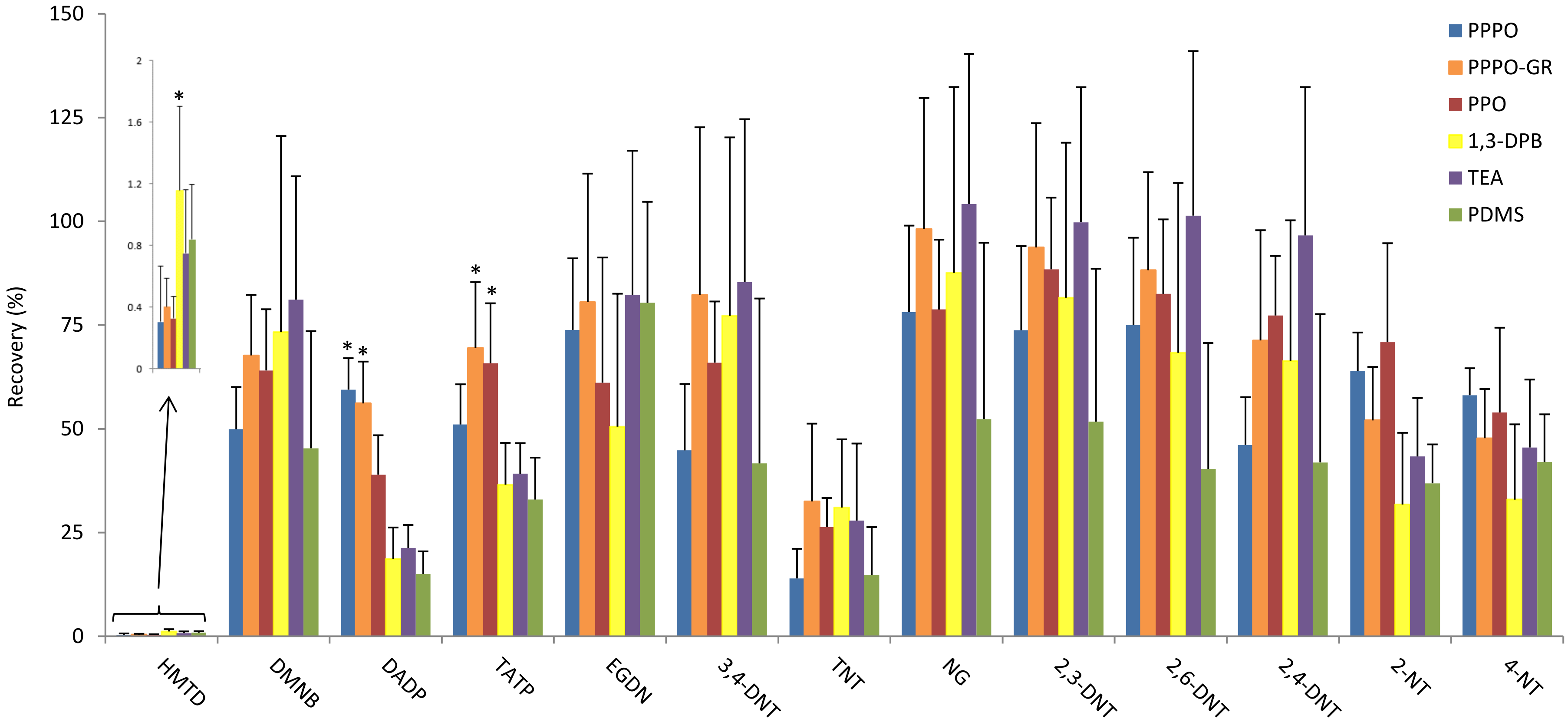

* Sorbents with significantly higher recoveries for n=6 replicates.

Figure S1. Average recoveries (72-h passive uptake) for the selected analytes using Nomex coated with Tenax (PPPO), Tenax-graphite (PPPO-GR), polydimethylphenyleneoxide (PPO), 1,3-diphenoxybenzene (1,3-DPB), triethanolamine (TEA) and polydimethylsiloxane (PDMS). Error bars show the standard deviation for n=6 replicate samples.

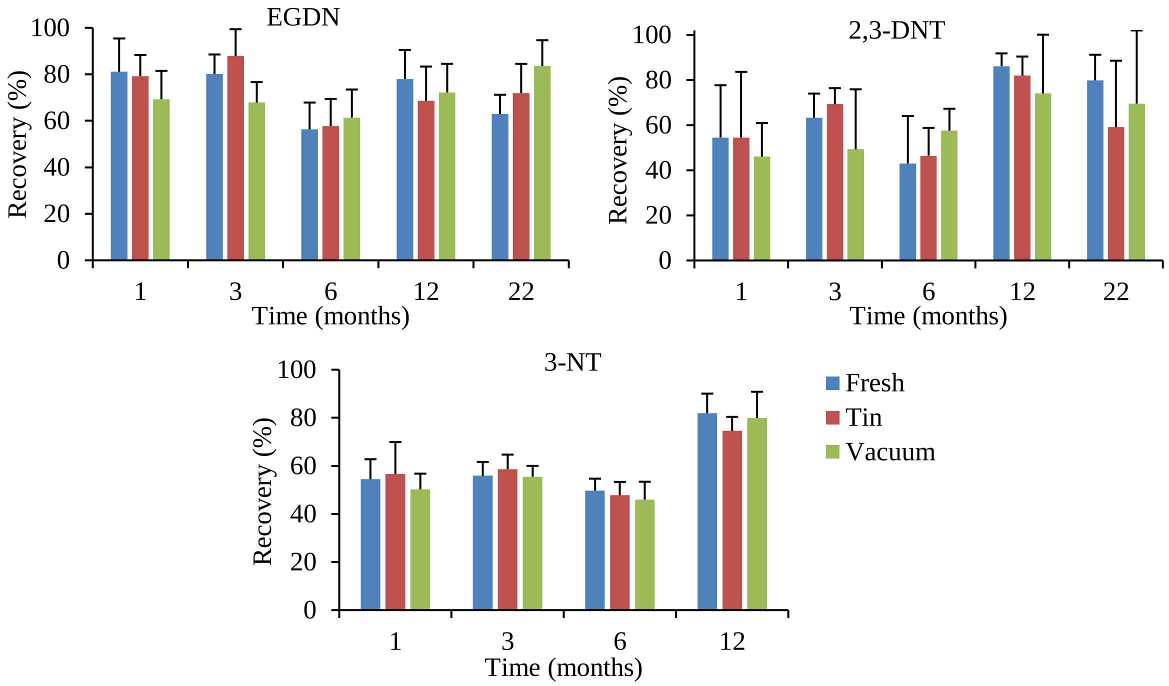

Figure S2. Passive uptake recoveries for three explosive components on samplers stored over time in a tin and under vacuum and compared to those freshly prepared. Sampler stability was measured over a 22-month period (except for 3-NT which was measured up to 12 months). Bars represent the average of n=6 replicates. Whiskers represent the standard deviation.

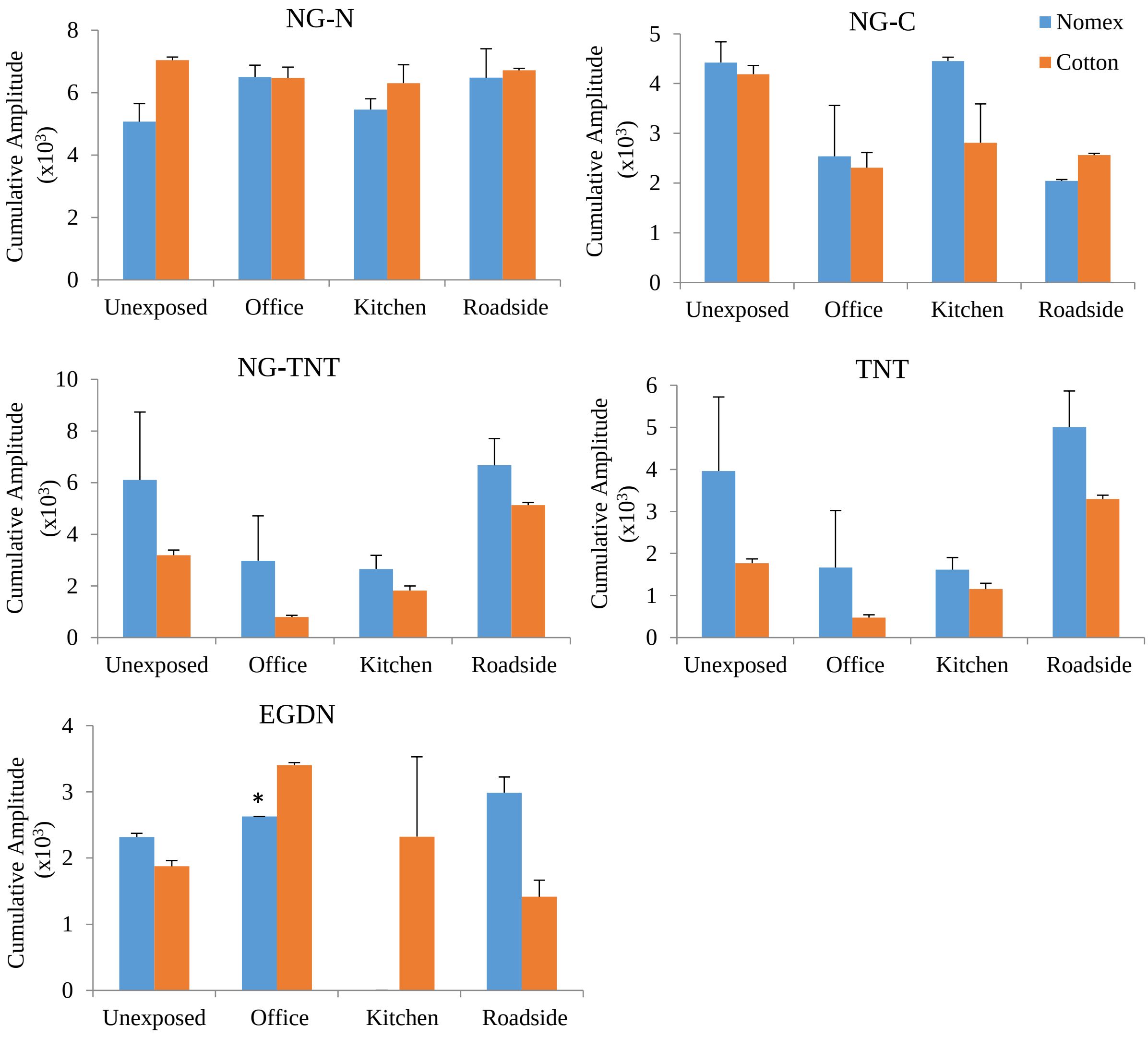

Figure S3. Assessment of interferences to analyte uptake on samplers following exposure to open environments using IMS analysis (n=2 samplers per exposure). Bars and whiskers represent the average and maximum/minimum value. The bar marked with * represents the only 1 positive result measured from the 2 samplers exposed for EGDN (cumulative amplitude for negative results below IMS ‘positive’ threshold not available to reliably calculate average for n=2 including the negative result).

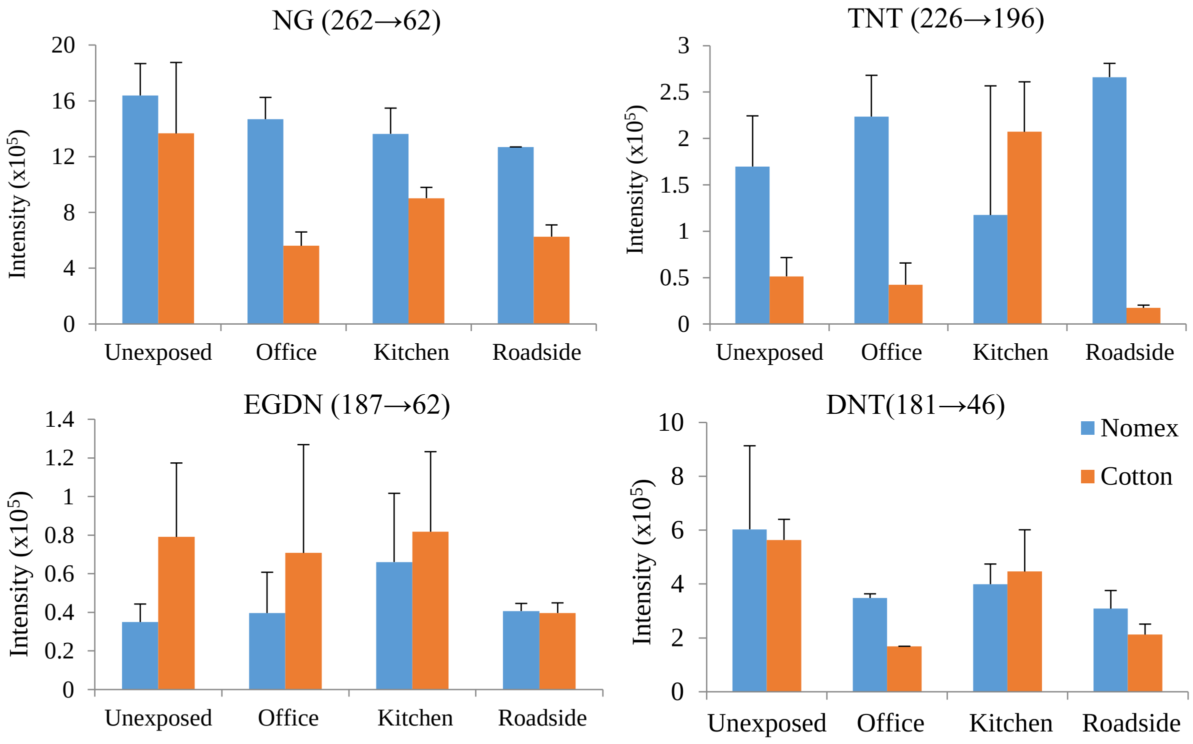

Figure S4. Assessment of interferences to analyte uptake on samplers following exposure to several simulated environments using TD-MS analysis (n=2 samplers per exposure). Bars and whiskers represent the average and maximum/minimum values, respectively.

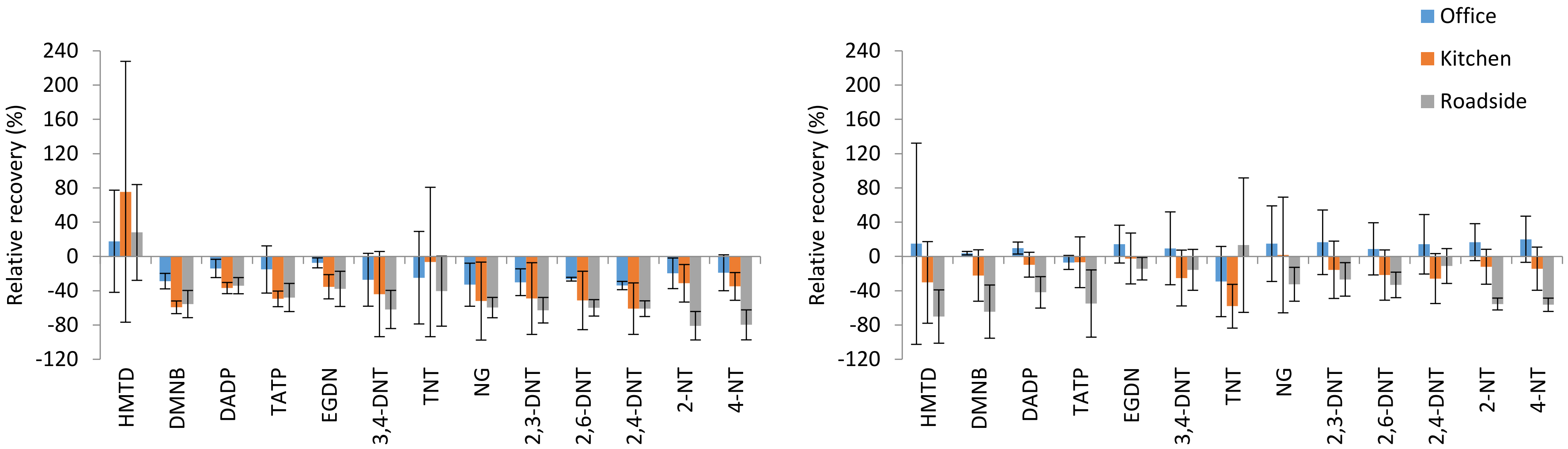

Figure S5. Assessment of analyte uptake on Nomex and cotton samplers following exposure to open environments using LC-HRMS analysis (n=3 samplers per exposure).

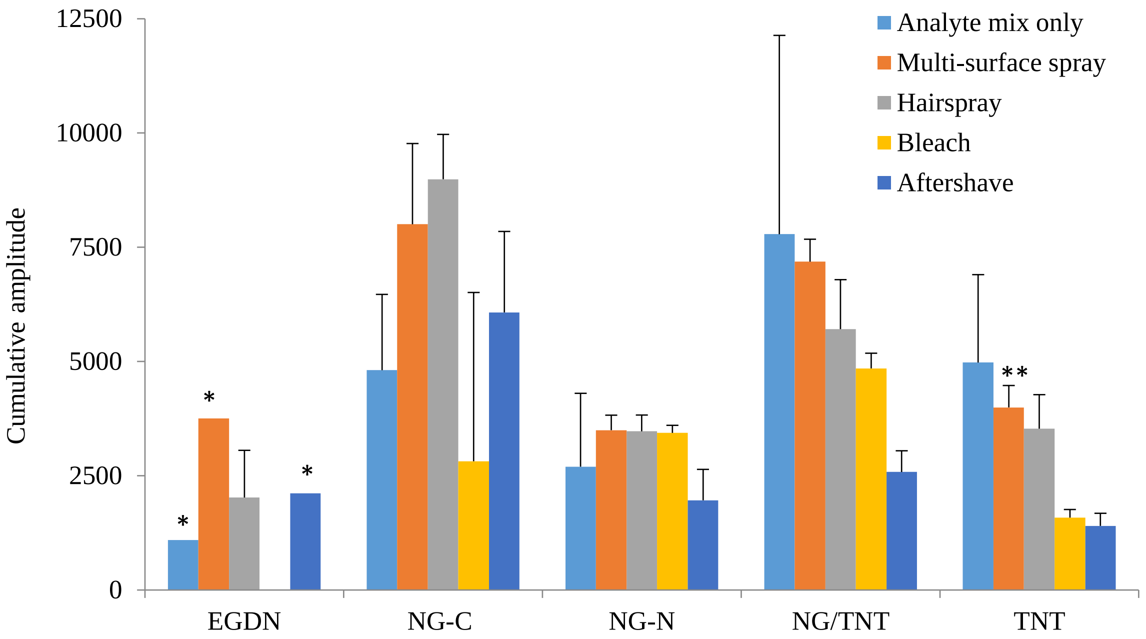

Figure S6. Assessment of analyte uptake on Nomex sampler when co-exposed with targeted interferences using IMS analysis (bars represent average for n=3 ±standard deviation unless otherwise marked). Bars marked with * represent values for only n=1 sampler of the three deployed which yielded a positive result for EGDN. Bar marked with ** represents the average value for n=2 samplers of the three deployed which yielded positive results for TNT. Cumulative amplitude for negative results below IMS ‘positive’ threshold were not available to reliably calculate an average for all n=3 replicates including the negative result(s).
